# Serodiagnosis and Risk Factors Associated with Infectious Agents of Reproductive Diseases in Bovines of Chiquinquirá, District of Boyacá (Colombia)

**DOI:** 10.1155/2022/7436651

**Published:** 2022-07-16

**Authors:** Deisy J. Lancheros-Buitrago, Diana M. Bulla-Castañeda, Martin O. Pulido-Medellin, Henry A. López Buitrago, Adriana M. Díaz-Anaya, Diego J. Garcia-Corredor

**Affiliations:** Veterinary Medicine and Zootechnology Research Group (GIDIMEVETZ), Universidad Pedagógica y Tecnológica de Colombia (UPTC), Tunja 15003, Colombia

## Abstract

The productivity of cattle farms is affected by infectious and noninfectious factors that generate economic losses and cause reproductive failure represented by low conception rates, embryonic mortality, abortions, and fetal mummification. The infectious agents that most impact the reproductive health of the bovine species from conception to birth are bovine herpesvirus type 1 (BoHV-1) causing infectious bovine rhinotracheitis (IBR), bovine viral diarrhea virus (BVDV), bovine parainfluenza virus type 3 (PI3), *Neospora caninum* and *Leptospira* spp. The objective of this study was to diagnose the presence of BoHV-1, bovine viral diarrhea (BVD), PI3, *Neospora caninum*, and *Leptospira* spp. by serology and identify the risk factors associated with infectious agents of reproductive interest in bovines of Boyacá (Colombia). A descriptive cross-sectional study was developed, with simple random sampling, where a sample size of 601 female cattle of Holstein, Jersey, and Normande breeds of different age groups was determined. Blood samples were taken and processed using the indirect ELISA technique (SYNBIOTICS®, SERELISA® BVD p80 Ab Mono Blocking, Ingezim R.12.NC.K, PRIMACHECK VPI-3®) and the MAT test for the diagnosis of bovine leptospirosis. The data were processed with the statistical program Epi Info™. The highest apparent seroprevalence was established for infectious bovine rhinotracheitis (61.1%), followed by BVD (37.6%), PI3 (40.9%), neosporosis (51.1%), and leptospirosis (14.8%). Variables such as age >4 years and Holstein breed for IBR and >4 years for BVD were established risk factors. Considering our results, we suggest implementing prevention and control plans that include vaccination as a prophylactic measure and biosecurity tools that reduce the probability of contagion and transmission of pathogens.

## 1. Introduction

The sustainability of a livestock farm begins with an adequate state of animal health, which depends mainly on the effective management of the multiple pathogens found in the farm [1]. In particular, the control of etiological agents of infectious diseases that generate significant negative impacts on the reproductive efficiency of cattle, both in the beef and dairy industry, as well as concomitant problems for human health and the environment, is due to the use of antimicrobials as a treatment for reproductive pathologies [2,3].

Infectious agents such as bacteria, viruses, protozoa, chlamydiae, and fungi are known to directly impact the reproductive health of cattle [4]. Bovine herpesvirus type 1 (BoHV-1), bovine viral diarrhea virus (BVDV), bovine parainfluenza virus type 3 (PI3), *Neospora caninum,* and *Leptospira* spp. are considered the main causative agent of reproductive disturbances in cattle around the world. Resulting in the presentation of clinical manifestations generated by the diseases infectious bovine rhinotracheitis (IBR), bovine viral diarrhea (BVD), bovine parainfluenza, bovine type 3 (PI3), neosporosis, and bovine leptospirosis, respectively [5–8]. The reproductive consequences of infection in an animal vary from outbreaks of abortion that can affect a large proportion of the pregnant herd to more subtle symptoms such as calving intervals, decrease in conception rate, and early embryonic mortality, among others that go unnoticed or undiagnosed [2]. The economic losses derived from reproductive events caused by infectious agents are high; therefore, proper reproductive efficiency management is essential for any livestock farm's economic success [9].

Considering the above impacts, prevention and control practices, preventive vaccination tools, and effective and efficient treatment protocols form the basis for preparing the immune system of each animal for possible exposure to pathogens on livestock farms. However, the implementation of other strategies focused on improving collective immunity through external factors such as nutrition, environment, and genetics also play a significant role in the management of animal production [1].

Based on the best available scientific information, effective recognition, prevention, and treatment of reproductive disorders are essential in the practice of veterinary professionals and farm owners. Thus, prevention and control protocols must be based on diagnostic tests that fit into the overall biosecurity production programs and must have measures to prevent pathogens' introduction and spread [2,3]. Therefore, the objective of this study was to evaluate serological evidence for the presence of infections caused by BoHV-1, BVD, PI3, *Neospora caninum*, and *Leptospira* spp. and to identify risk factors potentially associated with such infectious agents in cattle in Boyacá (Colombia).

## 2. Materials and Methods

### 2.1. Study Area

The study was conducted in the town of Chiquinquirá, located in the west of the department of Boyacá (latitude 5°36′48″ north, longitude 0°15′21″ meridian of Bogotá, 2000 to 3200 m.a.s.l, and mean temperature of 15°C). The township is the economic and commercial center of the western region of the department, where the agricultural sector relies on milk production and its derivatives [10].

### 2.2. Sample Size

The livestock census conducted by the Colombian Agricultural Institute - ICA reported that for the year 2020, Chiquinquirá had 31082 heads of cattle [11]. As a result, a sample size of 601 unvaccinated animals was determined with a confidence interval of 95% (CI) and an accepted error of 4% implementing the following equation:

(1) $$\begin{array}{c} n=\left(\frac{Z_{a}/2\sqrt{p\left(1-p\right)}}{E}\right)=\frac{Z^{2}\alpha /2\cdot p\left(1-p\right)}{E^{2}}, \end{array}$$

where *n* = sample size, *E* = accepted error, *p* = expected value of the proportion, and *α* = tail probability. Parallel to the sampling, an epidemiological survey was designed to collect information such as the age group and the breed of the animal and those reproductive events related to the diseases evaluated.

### 2.3. Sample Collection and Processing

Blood samples were collected by puncturing the coccygeal vein using the multiple needle gauge no. 21 g *x* 1 (Vacutainer®). Seven ml of biological material was collected and deposited in the BD Vacutainer® tubes without anticoagulant. The samples were refrigerated (4°C) and transported to the Veterinary Parasitology Laboratory of the Universidad Pedagógica *y* Tecnológica de Colombia. In the laboratory, the blood samples were centrifuged at 2500 r.p.m. for 10 minutes to separate the cells from the serum. Then, with a Pasteur pipette, the serum or the supernatant was transferred to the 1.5 ml Eppendorf type storage tube for storage at −20°C until testing [12].

An indirect enzyme-linked immunosorbent assay (ELISA) for the detection of specific antibodies against IBR, BVD, PI3, and neosporosis was carried out, using the commercial kits SYNBIOTICS® (sensitivity 96%; specificity 98%), SERELISA® BVD p80 Ab Mono Blocking, (sensitivity 98%; specificity 100%), PRIMACHECK VPI-3® (sensitivity 97%; specificity 99%), and Ingezim R.12NC.K1 (Ingenasa S.A) (sensitivity 70%; specificity 100%), by following the manufacturer's protocol. In addition, leptospirosis was diagnosed by the microscopic agglutination test (MAT); animals were considered to be positive when titers were ≥1 : 100 (sensitivity 60%; specificity 100%).

### 2.4. Statistical Analysis

An observational, descriptive, cross-sectional study was carried out with a simple random sampling of animals, where the sampling unit was female cattle. The apparent prevalence (AP) and the real prevalence (RP) were determined with the WinEpi statistical program. After consolidating and filtering the database, the data were analyzed using statistical program Epi Info® version 7.2.4.0. To estimate the prevalence ratio (PR), the proportion of animals and herds affected by the disease exposed to a factor was compared to the same proportion of the population not exposed to that factor. This PR was used to measure the association between reproductive agents and hypothesized causal factors, as well as the significance of these associations using Fisher's exact test [13]. The dependent variable included the serological results obtained; the independent variables were all considered in the epidemiological survey. Once these factors were established, logistic regression was performed to establish possible correlations.

### 2.5. Ethical Considerations

The study was conducted under the laws 576 of 2000 and 84 of 1989 of the Republic of Colombia. Informed consent was obtained from the owners of the specimens prior to sample collection.

## 3. Results

The apparent prevalence (AP) and the real prevalence (RP) of the infectious diseases were determined where seropositivity was 61.1% and 62.9% for IBR, respectively, (positive predictive value (PP+) 98.8% and negative predictive value (NP-) 93.8%), 37.6% and 38.4% for BVD, 40.9% and 41.6% for PI3, 51.1% and 73% for neosporosis, and 14.8% and 24.7% for bovine leptospirosis, respectively, (Table 1).

Regarding the age groups evaluated, individuals >4 years old presented the highest seroprevalence for IBR and BVD (68.9% and 50.6%, respectively). In contrast, for PI3, neosporosis, and leptospirosis, cattle 1–4 years old presented the highest seropositivity (Table 1). Concerning the breeds evaluated, Holstein presented the highest seroprevalence to IBR and PI3 and Jersey presented the highest seropositivity to *N. caninum*, while BVD and *Leptospira* spp. were more prevalent in cattle of the Normande breed (Table 2).

Statistical significance was established between the breeds and age groups evaluated through the presence of antibodies against IBR. Likewise, the presence of BVD was associated with the age groups of the cattle. Furthermore, a significant statistical association was determined between seropositivity to the disease and the reproductive events taken into account in the epidemiological survey. Cattle >4 years old, Holstein breed, AI, fetal death, cows with a history of inseminations without reproductive success, and noncertified semen were considered possible risk factors for IBR. In addition, individuals older than four years were established as possible risk factors for BVD occurrence. Natural mating and weak calves at birth presented the same condition but for neosporosis seropositivity (Table 3).

Logistic regression analysis identified that the age group >4 years and the Holstein breed were risk factors for IBR; furthermore, bovines with a history of fetal death in pregnancy, cattle with a history of inseminations without reproductive success, and dairy cows pregnant with uncertified semen could have 2.4383, 2.1222, and 2.9633 possibilities of being positive for the disease, respectively. For BVD, individuals older than 4 years were considered risk factors for the disease. On the other hand, cattle that are pregnant through natural mating and weak calves at birth may have 1.8936 and 2.6266 more possibilities of being seropositive for *Neospora* (Table 4).

## 4. Discussion

In this study, the disease with the highest seropositivity in the individuals evaluated was BoHV-1. Seroprevalences ranging from 35.65% to 73.13% are reported in Colombia [14–18]. On the other hand, diagnosis by serology for *Neospora* indicated that 51.1% of the cattle evaluated were seropositive, and these results are similar to national-level seroprevalences ranging from 2.8% to 57.5% [15,19–26]. Likewise, it has been reported that 40.9% of cattle had antibodies against PI3, a value that is in agreement with other studies performed in Colombia, which are between 11.20% and 88% [15,27–30]. It was demonstrated in this study that there is a wide distribution of seropositivity to IBR, BVD, PI3, leptospirosis, and neosporosis in Chiquinquirá, accompanied by a high transmission of these pathogens.

Regarding BVD, the values determined in the present study are 37.6%, which are between the ranges reported for the disease, which range from 29.7% to 76.4% [8,15,16,21,23,31]. Considering the seroprevalence determined for leptospirosis, 14.8% is a lower value than those established nationally, between 23.1% and 74.5% for the disease [16,20,23,32–34]. The findings are considered due to the risk of animal infection by *Leptospira* spp. associated with environmental factors such as rainfall and adverse climate, management factors, and farming systems such as cobreeding with other productive species or the presence of domestic animals (canines) and wild animals (rodents) in the herd [35].

Similarly, it is relevant to note that cattle are commonly grouped in herds, where prevalence estimates depend on the sampling strategy; thus, the presence of antibodies may vary from one investigation to another [36]. Hence, a limitation of this type of study is the ELISA that is intended for use as an aid in identifying animals with an adaptative immune response, indicating recent or prior infection. This has been reported by Kipyego et al. [37] in bovine herpesvirus type 1; however, the cattle sampled had no history of vaccination.

Cattle older than four years presented the highest seropositivity to BVDV, which is in agreement with previous studies. González-Bautista et al. [8] found the highest seroprevalence for this same age group in Sotaquirá. In Chiquinquirá, a significant statistical association was found between IBR seropositivity, breeds, and age groups evaluated, which differs from those reported by Doria-Ramos et al. [30], but is in agreement with the study conducted by Kipyego et al. [37], who reported a positive correlation with aging of cattle, where the odds of older dairy cattle being BoHV-1 antibody positivity which were 1.200 times higher with each additional year of age. Likewise, Doria-Ramos et al. and González-Bautista et al. [8,30] reported a statistically significant correlation between aging and certain breeds with seropositivity to BVDV, which is similar to the findings reported in this study.

Regarding PI3, neosporosis, and leptospirosis, cattle aged 1–4 years presented the highest seropositivity, which is not in agreement with the studies by Betancur et al. [27], Betancur et al. [32], Cardona et al., and Bedoya et al. [22,25], who found the highest seroprevalences for PI3, leptospirosis, and neosporosis, respectively, in older cattle, and this may occur because infected cattle remain infected for life; this effect is likely to represent the risk of cumulative exposure to the agent in the environment as the animals' age [37,38].

Holstein and Jersey cattle are the breeds that had the highest amount of antibodies against PI3 and neosporosis, which does not agree with Fernandez et al. [29], who indicated that the most prevalent breed for PI3 was Jersey and with the higher seropositivity of the Holstein breed found by Cruz-Estupiñan et al. [26]*;* however, this can occur due to the different management practices implemented on the farms and because these are dairy herds. No significant statistical association was obtained between PI3, leptospirosis, and neosporosis with the breed and age group of the cattle evaluated. This agrees with the findings of Fernandez et al. [29], Betancur et al. [32], Cruz-Estupiñan et al. [26], and Ansari-lari [7] but differs with Bedoya et al. [25], who found an association with age and established it as a risk factor for infection with *Neospora*. Doria-Ramos et al. [30] reported a relationship between the breed and age with the presentation of PI3; however, in Chiquinquirá, no association was found between seropositivity for antibodies against PI3 and the described variables.

In the present study, artificial insemination (AI) showed a significant association with seropositivity to BoHV-1 and leptospirosis. The transmission of pathogens occurs when there are no prior preventive measures in reproductive technologies; hence, mitigation of this risk should be considered during their development. Likewise, implementing biosecurity protocols and excellent quality control is imperative to prevent contamination and transmission of diseases while using these tools [39,40]. The quality of semen used to inseminate cattle is associated with the different diseases evaluated here. The use of noncertified semen, that is, semen freedom of these agents might become a risk factor for IBR, as previously reported [41–44]. There is significant evidence that these infections have other impacts on dairy cows, which are reflected in reduced conception rates, which is why the cows with a history of inseminations without reproductive success were considered as a risk factor for IBR appearance in the females evaluated.

Dystocic parturition, natural mating, fetal death, and weak calves at birth presented a significant statistical association with the seropositivity to neosporosis antibodies (*p* ≤ 0.05), and bovines with a history of fetal death in pregnancy, cattle with a history of inseminations without reproductive success, and dairy cows pregnant with uncertified semen have possibilities of being positive for the IBR. This is associated with what was reported by de Barros et al. [45] who mentioned that the correlations between the reproductive variables of seronegative animals were normal, while these relationships were weakened in seropositive animals.

## 5. Conclusion

High seroprevalences of antibodies against BoHV-1, BVDV, bovine parainfluenza virus type 3, *N. caninum y and Leptospira* spp. were found in bovine females in Chiquinquirá, Colombia, indicating the dissemination of pathogens in different herds and a high transmission level. Cattle older than 4 years presented the highest seroprevalence for BoHV-1 and BVDV, while cattle aged 1–4 years had higher seropositivity to PI3, neosporosis, and leptospirosis. Regarding breeds, Holstein showed the highest prevalence for BoHV-1 and PI3, Jersey for neosporosis, and Normande for BVD and leptospirosis. Bovine >4 years and Holstein breed for IBR and >4 years for BVD were considered as risk factors.

## Figures and Tables

**Table 1:** Prevalence of IBR, BVD, PI3, neosporosis, and leptospirosis in cattle in Chiquinquirá, Boyacá.

Disease	No of animals tested	Positive	AP (%)	RP (%)	PP + (%)	NP - (%)
IBR (BoHV-1)	601	367	61.1	62.9	98.8	93.8
BVD	601	225	37.6	38.4	100	98.8
PI3	601	245	40.9	41.6	98.6	97.9
Neosporosis	601	307	51.1	73	100	55.2
Leptospirosis	601	89	14.8	24.7	100	88.4

**Table 2:** Prevalence of IBR, BVD, PI3, neosporosis, and leptospirosis by the age group and the breed in cattle in Chiquinquirá, Boyacá.

Disease	Category	No of animals tested	Positive	Seropositive (%)
Age group				

IBR (BoHV-1)	<1 year	106	54	50.9
	1–4 years	183	98	53.6
	>4 years	312	215	68.9

BVD	<1 year	106	19	17.9
	1–4 years	183	49	26.8
	>4 years	312	158	50.6

PI3	<1 year	106	43	40.6
	1–4 years	183	80	43.7
	>4 years	312	123	39.4

Neosporosis	<1 year	106	51	48.1
	1–4 years	183	101	55.2
	>4 years	312	155	49.7

Leptospirosis	<1 year	106	11	10.4
	1–4 years	183	32	17.5
	>4 years	312	46	14.7

Breed				

IBR (BoHV-1)	Holstein	427	282	66.0
	Jersey	60	29	48.3
	Normande	114	54	47.4

BVD	Holstein	427	160	37.5
	Jersey	60	22	36.7
	Normande	114	44	38.6

PI3	Holstein	427	179	41.9
	Jersey	60	20	33.3
	Normande	114	47	41.2

Neosporosis	Holstein	427	211	49.4
	Jersey	60	35	58.3
	Normande	114	61	53.5

Leptospirosis	Holstein	427	71	16.6
	Jersey	60	7	11.7
	Normande	114	21	18.4

**Table 3:** Possible risk factors associated with IBR, BVD, PI3, neosporosis, and leptospirosis infections and results are shown as the prevalence ratio (PR) and the 95% confidence interval (95% CI). Significance is denoted by a value of *p* < 0.05.

Disease	Variable	Category	PR	CI (95%)	*p* value
IBR (BoHV-1)	Age group	<1 year	0.7495	0.5980–0.9394	0.012894936
		1–4 years	0.7674	0.6271–0.9394	0.008220621
		>4 years	1.5248	1.2421–1.8718	0.000289377
	Breed	Holstein	1.5617	1.2850–1.8978	0.000153039
		Jersey	1.7263	0.5556–0.9493	0.02413266
		Normande	0.6789	0.5497–0.8383	0.00069915

BVD	Age group	<1 year	0.7089	0.6311–0.7962	0.00013775
		1–4 years	1.7874	0.6983–0.8879	0.000170394
		>4 years	1.5493	1.3613–1.7632	0.00043

IBR (BoHV-1)	Reproductive events	Abortion	1.2214	0.9999–1.4921	0.032483287
		Artificial insemination (AI)	1.403	1.1440–1.7206	0.001440456
		Player loan	0.7348	0.6905–1.9143	0.006660251
		Fetal death	1.641	1.1960–2.2515	0.000443786
		Cows with a history of inseminations without reproductive success	1.4844	1.2167–1.810	0.000170702
		Retention of the placenta	0.8238	0.637–1.0225	0.043729409
		Certified semen	0.7202	0.5788–1.6157	0.005956667
		Uncertified semen	1.8092	1.2465–2.6259	0.000248774
		Shared bull	0.6337	0.5127–0.7831	0.000136516
BVD		Dystocia	0.7429	0.6603–1.0358	0.00019478
		Natural mating	0.7551	0.5489–1.0818	0.00000006
		Uncertified semen	0.929	0.8066–1.0700	0.000675
PI3		Natural mating	1.1383	0.9914–1.3070	0.046625492
Neosporosis		Dystocia	1.1924	0.9931–1.4318	0.031627136
		Natural mating	1.3268	1.1277–1.5610	0.00082658
		Fetal death	0.6609	0.7634–1.0752	0.00046591
		Weak calves at birth	1.7133	1.2691–2.3129	0.00309215
Leptospirosis		Dystocia	0.9386	0.8790–1.0023	0.046435523
		Artificial insemination	0.9085	0.8307–1.0937	0.014191688
		Certified semen	0.9136	0.8418–0.9916	0.013243401
		Uncertified semen	0.9247	0.8596–1.0947	0.049964738

**Table 4:** Analysis of different variables as possible risk factors for seropositivity to IBR, BVD, PI3, neosporosis, and leptospirosis infections in cattle in Chiquinquirá, Boyacá.

Disease	Variable	OR	Lower confidence interval (95%CI)	Upper confidence interval (95% CI)	*p* value
IBR	>4 years	1.8185	1.2845	2.5746	0.0007
	Holstein	1.4936	0.9572	2.3304	0.0772
	IA	0.7294	0.4425	1.2025	0.2161
	Fetal death	2.4383	1.5021	3.9581	0.0003
	Cows with a history of inseminations without reproductive success	2.1222	1.3943	3.2301	0.0004
	Uncertified semen	2.9633	1.6967	5.1755	0.0001

BVD	>4 years	3.3344	2.3476	4.736	0.0002

Neosporosis	Natural mating	1.8936	1.3109	2.7353	0.0007
	Weak calves at birth	2.6266	1.661	4.1537	0.0005

## Data Availability

The data used to support the research findings are included within the article.
